# Percutaneous nephrostomy as a marker of clinical vulnerability in non-metastatic muscle-invasive bladder cancer: prognostic and infectious implications

**DOI:** 10.3389/fonc.2026.1820805

**Published:** 2026-05-29

**Authors:** İlkay Çıtakkul, Yasemin Bakkal Temi, Ece Baydar, Elif Şahin, Umut Kefeli, Devrim Çabuk, Kazım Uygun

**Affiliations:** 1 Department of Internal Medicine and Medical Oncology, Kocaeli University, İzmit, Kocaeli, Türkiye; 2 Department of Medical Oncology, Kocaeli City Hospital, İzmit, Kocaeli, Türkiye

**Keywords:** hydronephrosis, infectious complications, muscle-invasive bladder cancer, overall survival, percutaneous nephrostomy

## Abstract

**Background:**

Percutaneous nephrostomy (PCN) is frequently employed to relieve malignant ureteral obstruction in patients with muscle-invasive bladder cancer (MIBC), yet its prognostic and infectious implications in non-metastatic disease remain poorly characterized. This study aimed to evaluate the association between PCN at diagnosis and clinical outcomes in patients with non-metastatic MIBC.

**Methods:**

We retrospectively analyzed 199 patients with non-metastatic MIBC treated between January 2019 and March 2025 at two centers in Türkiye. Patients were grouped by PCN status at diagnosis. The primary endpoint was overall survival (OS); secondary endpoints were metastasis-free survival (MFS) and infection-related outcomes. Survival was analyzed using Kaplan–Meier and multivariable Cox regression, including ECOG performance status and treatment modality as covariates. Logistic regression was used to assess infectious outcomes. Collinearity was evaluated using variance inflation factor analysis.

**Results:**

PCN was present in 27.1% of patients. Median OS was shorter in the PCN group compared with those without PCN (24 vs. 41 months; p = 0.004); however, PCN did not retain independent prognostic significance in the multivariable model after adjustment for ECOG performance status, CRP, N stage, and treatment modality (HR 0.433; 95% CI 0.175–1.068; p = 0.069). ECOG performance status (HR 1.819; p = 0.039), CRP (HR 1.005; p = 0.01), and N stage (HR 1.724; p = 0.023) were independent predictors of OS. A subgroup analysis within the hydronephrosis-positive population showed no significant difference in OS between PCN and non-PCN patients (26 vs. 29 months; p = 0.785). Treatment modality was the sole independent predictor of MFS (HR 0.594; p = 0.008). PCN was a strong and independent predictor of positive urine cultures (OR 2.685; 95% CI 1.055–6.837; p = 0.038) and infection-related hospitalizations (OR 13.995; 95% CI 4.923–39.785; p < 0.001).

**Conclusions:**

In patients with non-metastatic MIBC, PCN was not an independent predictor of OS after comprehensive adjustment, suggesting that the survival disadvantage observed in unadjusted analysis reflects baseline clinical vulnerability rather than a direct effect of the procedure. PCN was, however, independently and strongly associated with infectious morbidity. These findings position PCN as a marker of clinical frailty that warrants careful multidisciplinary evaluation before placement and proactive management of infectious complications thereafter.

## 1 Introduction

Bladder cancer is among the most common urological malignancies worldwide, and muscle-invasive bladder cancer (MIBC) carries a substantially worse prognosis than non-muscle-invasive disease (1, 2). Accurate staging, timely intervention, and appropriate systemic therapy are central to improving outcomes in this population.

Hydronephrosis in patients with MIBC typically reflects ureteral obstruction or direct tumor invasion and has been consistently associated with worse overall and cancer-specific survival in multiple studies (3–7). Beyond its anatomical significance, hydronephrosis carries clinical weight in treatment planning: it may affect eligibility for neoadjuvant chemotherapy, influence the feasibility of radical cystectomy, and inform decisions regarding bladder-preserving chemoradiotherapy (8).

Percutaneous nephrostomy (PCN) is one of the most widely used interventions for relieving upper urinary tract obstruction in this setting. While PCN addresses the immediate problem of obstruction, it introduces its own set of complications — most notably an elevated risk of infectious morbidity, potential delays in treatment initiation, and difficulties in maintaining access to systemic therapy (9–12). Patients who undergo PCN tend to present with more advanced disease features, have reduced access to neoadjuvant chemotherapy, and experience higher rates of infection-related complications compared with those who do not (9, 11, 13, 16, 17). Despite this, the evidence base specifically addressing the prognostic and infectious implications of PCN in non-metastatic MIBC remains sparse. Most available data come from studies that include metastatic or heterogeneous cancer populations, limiting the applicability of their findings to patients treated with curative intent (12–15, 23).

Against this background, the primary objective of this study was to evaluate the association between PCN at diagnosis and overall survival in a well-defined cohort of patients with non-metastatic MIBC. The secondary objectives were to assess its relationship with metastasis-free survival and infection-related outcomes, including positive urine cultures and infection-related hospitalizations.

## 2 Materials and methods

### 2.1 Study design and participants

This retrospective dual-center cohort study included consecutive patients diagnosed with non-metastatic MIBC between January 2019 and March 2025 at the Kocaeli University Hospital Oncology Center and Kocaeli City Hospital. Eligible patients were adults (≥18 years) with histologically confirmed MIBC established by transurethral resection of the bladder tumor (TURBT) following diagnostic cystoscopy. Clinical staging and assessment to rule out metastatic disease were conducted using cross-sectional imaging of the abdomen, pelvis, and thorax, including computed tomography (CT) and magnetic resonance imaging (MRI) scans. The presence of hydronephrosis was assessed using cross-sectional imaging techniques, specifically ultrasound or CT, at the time of initial diagnosis. For statistical analysis, hydronephrosis was classified as either present or absent. Patients with non–muscle-invasive disease, small cell histology, or metastatic disease on baseline imaging were excluded.

### 2.2 Data collection and definitions

Electronic medical records were systematically reviewed to gather demographic, clinical, and pathological variables, including age, sex, comorbidities, ECOG performance status, tumor characteristics, and treatment modalities (radical cystectomy, neoadjuvant chemotherapy, and bladder-preserving approaches). Tumor responses were assessed according to the RECIST version 1.1 (18). The estimated glomerular filtration rate (eGFR) was calculated using the Chronic Kidney Disease Epidemiology Collaboration (CKD-EPI) equation. Renal dysfunction was defined as an eGFR of less than 60 mL/min/1.73 m², in line with established chronic kidney disease criteria (19, 20). Additional variables of interest included the presence of PCN, positive urine cultures during follow-up, and infection-related hospitalization. PCN status was assessed at diagnosis, before the initiation of any curative therapy. PCN placement was determined at each participating center through multidisciplinary clinical evaluation based on individual patient indications, such as hydronephrosis-related renal dysfunction or symptomatic upper tract obstruction.

A positive urine culture was defined as the growth of ≥10^5^ CFU/mL of a single uropathogen (21, 25). In the PCN group, samples were obtained aseptically from the nephrostomy tube during hospitalization. In patients without a nephrostomy, urine cultures were collected by midstream clean-catch in non-catheterized patients, or from the urinary catheter in catheterized patients, under clinical indications. A clinically significant infection was defined as hospitalization due to a urinary tract infection necessitating intravenous antimicrobial therapy or medical intervention, in order to distinguish colonization and asymptomatic bacteriuria from clinically meaningful infectious events (21, 25).

### 2.3 Primary and secondary outcomes

The primary outcome measure was overall survival (OS), defined as the duration from diagnosis to death from any cause. Secondary outcomes included metastasis-free survival (MFS) and infection-related event rates. MFS was defined as the interval from diagnosis to the first radiologically confirmed distant metastasis or death, whichever occurred first. Patients who did not experience any events were censored at the time of their last clinical follow-up. For the purposes of survival analysis, treatment modality was categorized as surgery-based (with or without neoadjuvant chemotherapy) or non-surgery-based. The non-surgery-based category included patients who received definitive chemoradiotherapy (n = 79) and those who developed metastatic disease during neoadjuvant chemotherapy and therefore did not proceed to curative-intent local treatment (n = 7).

### 2.4 Statistical analysis

All statistical analyses were conducted using IBM SPSS Statistics for Windows version 29.0 (IBM Corp., Armonk, NY, USA). The Kolmogorov–Smirnov and Shapiro–Wilk tests were applied to assess the assumption of normality. Continuous variables were presented as medians with interquartile ranges (IQR) and intergroup differences were assessed using the Mann–Whitney U test due to non-normal distribution. Clinical T stage was dichotomized as T2 versus T3–4, and clinical N stage as N0 versus N1–3, to ensure adequate cell counts and statistical stability in comparative and regression analyses. Categorical data were reported as frequencies and percentages and analyzed using the chi-square or Fisher’s exact test, depending on the expected cell counts.

The Kaplan–Meier method was employed to assess OS and MFS outcomes across groups, and group comparisons were performed using the log-rank test. To assess whether PCN carries independent prognostic significance beyond hydronephrosis, a subgroup analysis was additionally performed within the hydronephrosis-positive population. Additionally, a PCN × hydronephrosis interaction term was incorporated into the multivariable Cox regression model for overall survival to examine whether the prognostic effect of PCN was modified by hydronephrosis status. Independent prognostic indicators for OS and MFS were evaluated using Cox proportional hazards regression. The multivariable Cox regression model included ten covariates — age, GFR, albumin, CRP, T stage, N stage, hydronephrosis, nephrostomy, ECOG performance status, and treatment modality — all of which were specified *a priori* based on established clinical relevance in MIBC. No stepwise selection or *post-hoc* variable removal was performed; all pre-specified variables were retained in the final model. Collinearity between covariates was assessed using variance inflation factor (VIF) analysis within a linear regression framework; all VIF values were below 5, indicating acceptable levels of collinearity. Results are presented as hazard ratios (HRs) with 95% confidence intervals (CIs).

Factors associated with infectious outcomes, including positive urine cultures and infection-related hospitalizations, were evaluated using binary logistic regression analysis. Results are reported as odds ratios (ORs) with 95% confidence intervals (CIs). In both Cox and logistic regression analyses, a p-value < 0.05 was considered statistically significant. For logistic regression models, model fit was assessed using the Hosmer–Lemeshow goodness-of-fit test, and discriminative performance was evaluated using the area under the receiver operating characteristic curve (AUC).

## 3 Results

### 3.1 Baseline characteristics of the cohort

The cohort comprised 199 patients with a confirmed diagnosis of MIBC. The cohort consisted of 87.9% men (n=175) and 12.1% women (n=24), with a median age of 66 years (IQR, 61–72). The Eastern Cooperative Oncology Group (ECOG) performance status was 0 in 63.8% (n=127) and 1–2 in 36.2% (n=72) of the patients. Histopathological examination revealed urothelial carcinoma in 98% of the patients (n=195), whereas 2% (n=4) exhibited squamous cell histology. The baseline demographic, clinical, and pathological characteristics are presented in **Table 1**.

**Table 1:** Baseline clinical and demographic characteristics and comparison of patients with and without percutaneous nephrostomy (PCN).

Characteristic	All patients (n=199)	PCN (+) (n=54)	PCN (–) (n=145)	p-value
Age (y), median (IQR)	66 (IQR 61–72)	67.5 (IQR 61–74)	66 (IQR 61–72)	0.36
Sex, n (%)				
Male	175 (87.9)	45 (83.3)	130 (89.7)	0.223
Female	24 (12.1)	9 (16.7)	15 (10.3)	
ECOG Performance Status, n (%)				
0	127 (63.8)	31 (57.4)	96 (66.2)	0.251
1–2	72 (36.2)	23 (42.6)	49 (33.8)	
Histology, n (%)				
Urothelial	195 (98.0)	50 (94.3)	145 (99.3)	
Squamous	4 (2.0)	3 (5.7)	1 (0.7)	
Clinical T Stage, n (%)				
T2	150 (75.4)	38 (70.4)	112 (77.2)	0.317
T3–4	49 (24.6)	16 (29.6)	33 (22.8)	
Clinical N Stage, n (%)				
N0	111 (55.8)	25 (46.3)	86 (59.3)	0.10
N1–3	88 (44.2)	29 (53.7)	59 (40.7)	
Hydronephrosis at diagnosis, n (%)	62 (31.2)	48 (88.9)	14 (9.7)	<0.001
CRP, median (IQR)	10 (3–29)	13 (5–36)	9 (2–22)	0.019
GFR, median (IQR)	65 (51–83)	51.5 (38–57)	72 (57–88)	<0.001
Albumin, median (IQR)	38 (28–43)	35 (22–41)	38 (28–43)	0.094
Treatment Modality, n (%)				
Surgery-based (± NAC)	113 (56.8)	27 (50.0)	86 (59.3)	0.139
Non-surgery based*	86 (43.2)	27 (50.0)	59 (40.7)	
ED visits, median (IQR)	1 (0–2)	2 (0–3)	0 (0–1)	<0.001

Percentages are calculated within each column.

*Includes 79 patients who received definitive chemoradiotherapy and 7 patients who developed metastatic disease during neoadjuvant chemotherapy.

PCN, percutaneous nephrostomy; ECOG, Eastern Cooperative Oncology Group; GFR, glomerular filtration rate; CRP, C-reactive protein; NAC, neoadjuvant chemotherapy; ED, emergency department; IQR, interquartile range.

Among the cohort, 113 patients (56.8%) received surgery-based treatment (with or without neoadjuvant chemotherapy), and 86 patients (43.2%) received non-surgery-based treatment, including 79 who received definitive chemoradiotherapy (CRT) and 7 who developed metastatic disease during neoadjuvant chemotherapy. PCN was performed in 54 patients (27.1%), whereas 145 patients (72.9%) did not undergo nephrostomy.

### 3.2 Comparison of patients with and without PCN

As shown in **Table 1**, patients with PCN demonstrated several clinically relevant differences compared with those without PCN. Group-wise comparison of ECOG performance status revealed no statistically significant variation (p = 0.251), and no clear pattern of higher scores was observed in either group. The median glomerular filtration rate (GFR) was significantly lower in the PCN group compared to those without PCN (51.5 vs. 72 mL/min/1.73 m², p < 0.001). Similarly, the median C-reactive protein (CRP) levels were significantly higher in patients with PCN (13 vs. 9 mg/L, p = 0.019). Regarding tumor burden, no significant difference was observed in the clinical T stage distribution (p = 0.317), and the clinical N stage also did not differ significantly between groups (p = 0.100). The incidence of hydronephrosis was significantly higher in patients with PCN, observed in 88.9% of cases, compared to 9.7% in those without PCN (p < 0.001). Albumin levels were numerically lower in the PCN group (median 35 vs. 38 g/L), though this difference did not reach statistical significance (p = 0.094). Other variables, including age (p = 0.36), sex distribution (p = 0.223), and treatment modality (p = 0.139), did not differ significantly between the groups.

### 3.3 Survival outcomes

**Figure 1:**
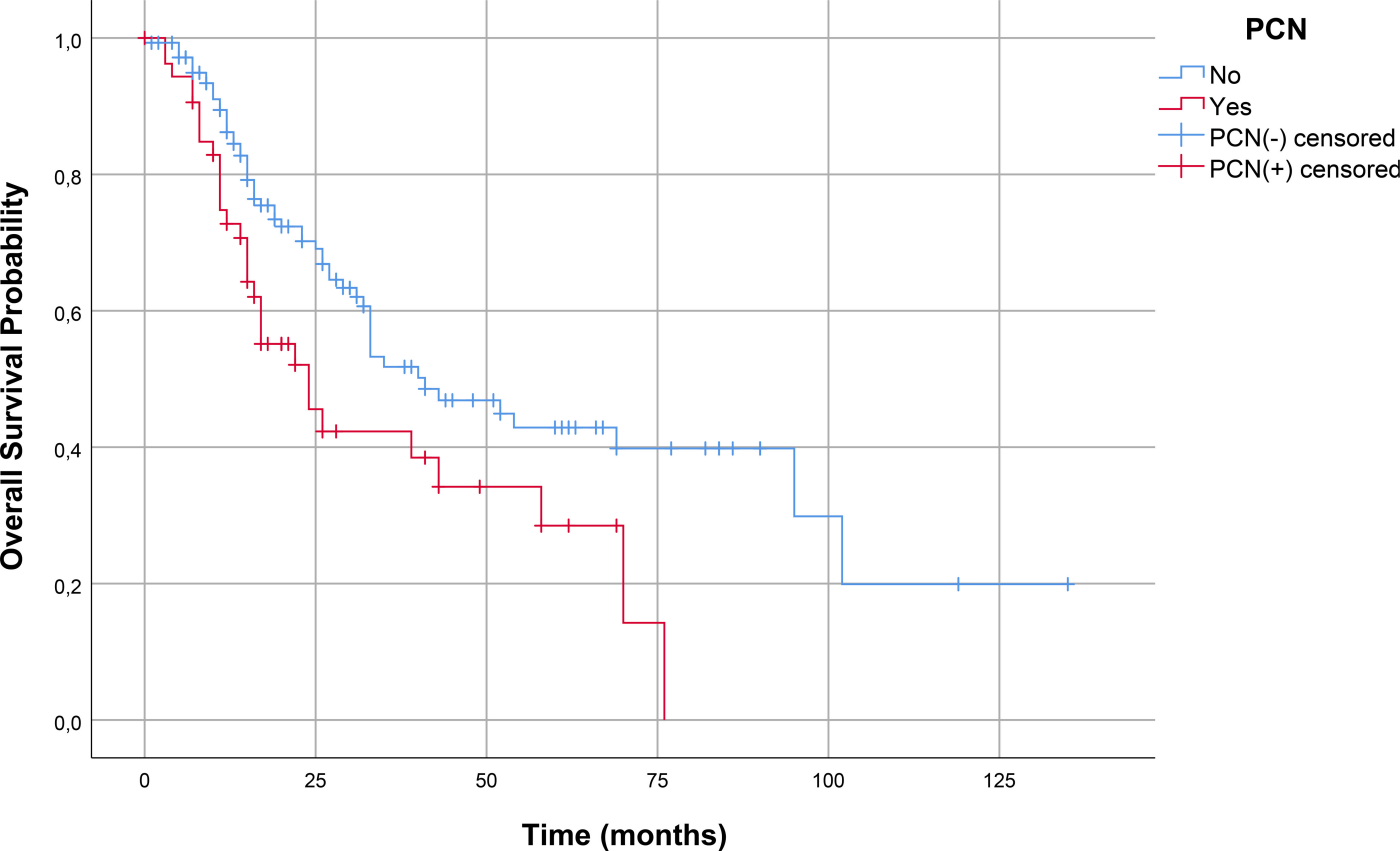
The median OS was 41.0 months (95% CI: 27.3–54.6) in the non-nephrostomy group and 24.0 months (95% CI: 15.3–32.6) in the nephrostomy group (p = 0.004). CI, confidence interval; OS, overall survival.

**Figure 2:**
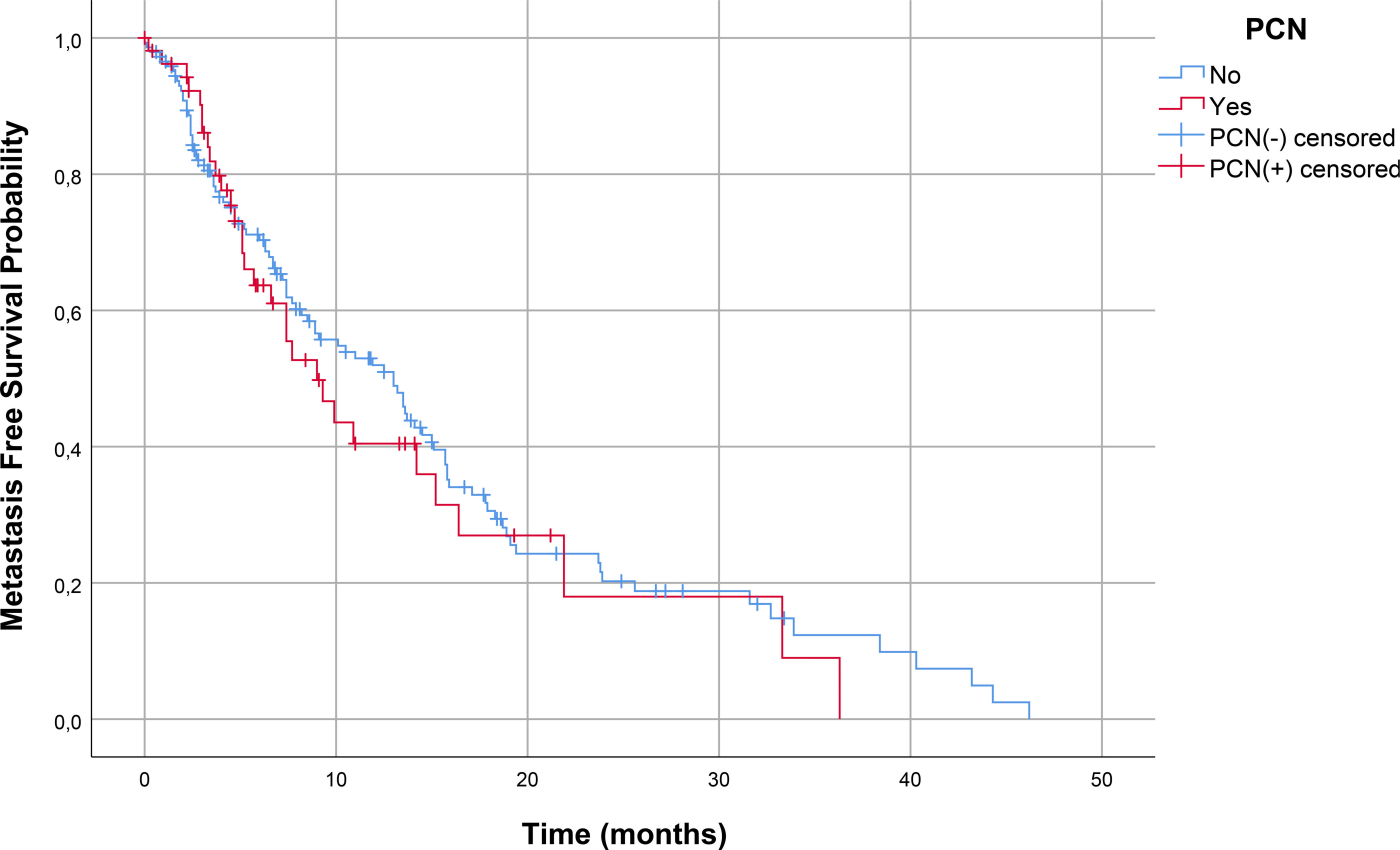
The median MFS was 13.0 months (95% CI: 10.6–15.3) in the non-nephrostomy group and 9.0 months (95% CI: 7.8–10.1) in the nephrostomy group (p = 0.55). CI, confidence interval; MFS, metastasis-free survival.

Kaplan–Meier analysis demonstrated that the median OS was 41.0 months (95% CI: 27.3–54.6) in the non-nephrostomy group and 24.0 months (95% CI: 15.3–32.6) in the nephrostomy group (log-rank p = 0.004) (**Figure 1**). The median MFS was 13.0 months (95% CI: 10.6–15.3) in the non-nephrostomy group and 9.0 months (95% CI: 7.8–10.1) in the nephrostomy group (log-rank p=0.55), with no significant difference between groups (**Figure 2**). To assess whether PCN holds prognostic significance independent of hydronephrosis, a subgroup analysis was conducted, focusing on patients diagnosed with hydronephrosis (n=62; PCN(+) n=48, PCN(–) n=14). Within this subgroup, characterized by the presence of hydronephrosis, the median OS was 26.0 months (95% CI: 2.6–49.4) for the PCN group and 29.0 months (95% CI: 17.4–40.6) for the non-PCN group. The difference between these groups was not statistically significant (log-rank p = 0.785). Additionally, a PCN × hydronephrosis interaction term was incorporated into the multivariable Cox regression model for overall survival to examine whether the prognostic effect of PCN was modified by hydronephrosis status. The interaction term was statistically significant (HR 0.194; 95% CI 0.044–0.856; p = 0.030), indicating that the prognostic effect of PCN on overall survival differed according to hydronephrosis status. This finding is consistent with the subgroup analysis, in which no significant survival difference was observed between PCN and non-PCN patients within the hydronephrosis-positive subgroup.

In the multivariate Cox regression analysis for OS, ECOG performance status (HR 1.819; 95% CI 1.030–3.211; p = 0.039), CRP levels (HR 1.005; 95% CI 1.001–1.008; p = 0.010), and N stage (HR 1.724; 95% CI 1.080–2.752; p = 0.023) emerged as independent predictors. Nephrostomy did not maintain independent prognostic significance (HR 0.433; 95% CI 0.175–1.068; p = 0.069). Similarly, hydronephrosis did not exhibit independent prognostic significance for OS (HR 1.597; 95% CI 0.654–3.903; p = 0.304). Concerning MFS, no variable achieved independent prognostic significance except for treatment modality (HR 0.594; 95% CI 0.405–0.872; p = 0.008) (**Table 2**).

**Table 2:** Cox regression analyses of predictors for overall survival and metastasis-free survival.

Variable	HR	95% CI	p-value	HR	95% CI	p-value
Overall Survival						
Univariate				Multivariate		
Age	1.019	0.992 – 1.047	0.16	1.000	0.970 – 1.030	0.988
GFR	0.990	0.981 – 0.999	0.02	0.996	0.987 – 1.007	0.495
Albumin	0.990	0.977 – 1.004	0.15	0.999	0.982 – 1.016	0.864
CRP	1.004	1.001 – 1.007	0.01	1.005	1.001 – 1.008	0.01
T stage (T3–4 vs T2)	1.303	0.794 – 2.136	0.295	1.165	0.659 – 2.059	0.599
N stage (N1–3 vs N0)	1.736	1.139 – 2.646	0.010	1.724	1.080 – 2.752	0.023
Hydronephrosis	1.525	0.982 – 2.367	0.06	1.597	0.654 – 3.903	0.304
Nephrostomy	1.892	1.215 – 2.946	0.005	0.433	0.175 – 1.068	0.069
ECOG (1–2 vs 0)	2.169	1.391 – 3.382	0.001	1.819	1.030 – 3.211	0.039
Treatment Modality (non-surgery vs surgery)	1.662	1.081 – 2.554	0.021	0.764	0.466 – 1.253	0.286
Metastasis-Free Survival						
Univariate				Multivariate		
Age	1.009	0.978 – 1.041	0.58	0.990	0.964 – 1.017	0.477
GFR	0.999	0.990 – 1.007	0.78	0.997	0.989 – 1.006	0.525
Albumin	1.004	0.987 – 1.022	0.62	0.993	0.979 – 1.006	0.275
CRP	1.002	0.999 – 1.006	0.18	0.998	0.994 – 1.002	0.309
T stage (T3–4 vs T2)	0.996	0.657 – 1.510	0.984	0.922	0.580 – 1.466	0.733
N stage (N1–3 vs N0)	0.949	0.669 – 1.347	0.770	1.115	0.755 – 1.645	0.584
Hydronephrosis	1.315	0.812 – 2.128	0.26	1.305	0.752 – 2.266	0.344
Nephrostomy	1.160	0.699 – 1.923	0.56	0.698	0.385 – 1.265	0.236
ECOG (1–2 vs 0)	1.300	0.894 – 1.891	0.169	1.682	0.994 – 2.846	0.053
Treatment Modality (non-surgery vs surgery)	0.701	0.493 – 0.998	0.049	0.594	0.405 – 0.872	0.008

HR, hazard ratio; CI, confidence interval; GFR, glomerular filtration rate; CRP, C-reactive protein; ECOG, Eastern Cooperative Oncology Group performance status; OS, overall survival; MFS, metastasis-free survival.

#### 3.3.1 Microbial growth in urine cultures

Univariate analysis revealed that microbial growth in urine cultures was significantly associated with elevated CRP levels (OR: 1.008; 95% CI: 1.000–1.016; p = 0.04), albumin levels (OR: 0.690; 95% CI: 0.612–0.778; p < 0.001), and the presence of nephrostomy (OR: 4.905; 95% CI: 2.510–9.585; p < 0.001). In the multivariate logistic regression model, elevated CRP levels (OR: 1.028; 95% CI: 1.010–1.047; p = 0.003) and the presence of nephrostomy (OR: 2.685; 95% CI: 1.055–6.837; p = 0.038) were identified as independent predictors of microbial growth in urine cultures. The findings are summarized in **Table 3**. The model demonstrated acceptable fit (Hosmer–Lemeshow p = 0.749), with an AUC of 0.706, indicating acceptable discriminative performance.

**Table 3:** Logistic regression analyses of predictors for positive urine culture and infection-related hospitalization.

Positive urine culture						
	Univariate			Multivariate		
Variable	OR	95% CI	p-value	OR	95% CI	p-value
Age	0.995	0.963 – 1.027	0.74	1.026	0.980 – 1.074	0.269
GFR	0.996	0.985 – 1.008	0.55	1.002	0.983 – 1.020	0.863
Albumin	0.690	0.612 – 0.778	<0.001	1.026	0.951 – 1.106	0.515
CRP	1.008	1.000 – 1.016	0.04	1.028	1.010 – 1.047	0.003
T stage (T3–4 vs T2)	1.016	0.429 – 2.405	0.972	1.374	0.497 – 3.797	0.538
N stage (N1–3 vs N0)	0.774	0.398 – 1.502	0.448	0.729	0.339 – 1.567	0.418
Nephrostomy	4.905	2.510 – 9.585	<0.001	2.685	1.055 – 6.837	0.038
Infection-Related Hospitalization						
	Univariate			Multivariate		
Variable	OR	95% CI	p-value	OR	95% CI	p-value
Age	0.987	0.947 – 1.028	0.51	1.020	0.972 – 1.070	0.424
GFR	0.986	0.972 – 1.001	0.06	1.020	0.998 – 1.042	0.076
Albumin	0.989	0.967 – 1.011	0.26	0.982	0.952 – 1.012	0.227
CRP	1.008	1.001 – 1.015	0.02	1.006	0.998 – 1.015	0.161
T stage (T3–4 vs T2)	1.075	0.451 – 2.559	0.871	1.913	0.643 – 5.691	0.243
N stage (N1–3 vs N0)	2.038	0.963 – 4.314	0.063	0.498	0.208 – 1.189	0.116
Nephrostomy	12.536	5.438 – 28.898	<0.001	13.995	4.923 – 39.785	<0.001

OR, odds ratio; CI, confidence interval; GFR, glomerular filtration rate; CRP, C-reactive protein; ECOG, Eastern Cooperative Oncology Group performance status.

#### 3.3.2 Infection-related hospitalization

In the univariate analysis, infection-related hospitalization was significantly associated with elevated CRP levels (OR: 1.008; 95% CI: 1.001–1.015; p = 0.02) and the presence of nephrostomy (OR: 12.536; 95% CI: 5.438–28.898; p < 0.001). In the multivariate analysis, only the presence of nephrostomy (OR: 13.995; 95% CI: 4.923–39.785; p < 0.001) remained an independent predictor of infection-related hospitalization. The findings are summarized in **Table 3**. The model demonstrated good fit (Hosmer–Lemeshow p = 0.770), with an AUC of 0.818, indicating good discriminative performance.

## 4 Discussion

Percutaneous nephrostomy is a frequently encountered clinical scenario in patients with non-metastatic MIBC, yet its prognostic and infectious implications in this specific population have received limited systematic attention. In our cohort of 199 patients, PCN was present in 27.1% at diagnosis and was strongly concentrated among patients with hydronephrosis, impaired renal function, elevated inflammatory markers, and more advanced nodal disease. When these baseline differences were accounted for in a multivariable model that included ECOG performance status, treatment modality, nodal stage, CRP, GFR, and albumin, PCN did not retain independent prognostic significance for overall survival (HR 0.433; 95% CI 0.175–1.068; p = 0.069). By contrast, PCN remained a robust independent predictor of both positive urine cultures (OR 2.685; 95% CI 1.055–6.837; p = 0.038) and infection-related hospitalizations (OR 13.995; 95% CI 4.923–39.785; p < 0.001), pointing to a clinically meaningful infectious burden that persists regardless of other patient characteristics.

The survival disparity between PCN and non-PCN patients was substantial in unadjusted analysis — a median OS of 24 versus 41 months (p = 0.004) — but this difference did not withstand full multivariable adjustment. The most likely explanation is that PCN, in this context, is not a biologic driver of poor survival but rather a marker of a clinically fragile patient profile. Patients who required nephrostomy had nearly universal hydronephrosis (88.9%), markedly reduced GFR (median 51.5 vs. 72 mL/min/1.73 m²), higher systemic inflammation, and more advanced nodal involvement. These are precisely the features that independently predicted worse OS in our model — ECOG performance status (HR 1.819; p = 0.039), CRP (HR 1.005; p = 0.010), and N stage (HR 1.724; p = 0.023). PCN itself, significant in univariate analysis (HR 1.892; 95% CI 1.215–2.946; p = 0.005), fell just short of the significance threshold after full adjustment (HR 0.433; 95% CI 0.175–1.068; p = 0.069). In this respect, PCN may be best understood as a clinical indicator that identifies a vulnerable subgroup rather than as an independent oncological determinant. The post-adjustment estimate of HR 0.433 for PCN should not be interpreted as evidence of a protective effect; the wide confidence interval crossing unity and the absence of biological plausibility suggest that this reversal most plausibly reflects negative confounding introduced by the strong correlation between PCN and the adjustment covariates — particularly hydronephrosis, GFR, and CRP — rather than a true protective association.

To examine whether PCN carries prognostic weight beyond hydronephrosis itself, a subgroup analysis was performed within the hydronephrosis-positive population (n = 62; PCN(+) n = 48, PCN(–) n = 14). Median OS was 26.0 months in the PCN group and 29.0 months in those without PCN, with no significant difference between groups (log-rank p = 0.785). The non-PCN hydronephrosis group was small, and the resulting confidence intervals were wide, so this comparison must be interpreted cautiously. Nevertheless, the absence of any survival difference within the hydronephrosis-positive subgroup is consistent with the view that PCN does not add independent prognostic information once the underlying obstructive disease is present. The significant PCN × hydronephrosis interaction (p = 0.030) further supports this interpretation, suggesting effect modification by hydronephrosis — that is, the prognostic role of PCN appears contingent on the presence of underlying obstructive disease rather than reflecting an independent effect of the procedure itself. In the main multivariable model, PCN showed a hazard ratio of 0.433 (95% CI 0.175–1.068; p = 0.069), falling just short of the conventional significance threshold. Given the modest cohort size, the possibility that a true association exists but lacked sufficient statistical power to emerge cannot be excluded. Whether PCN retains residual independent prognostic significance will require prospective evaluation in larger cohorts.

The pattern we observed — poor survival among PCN recipients driven largely by baseline clinical burden rather than the procedure itself — is broadly consistent with the existing literature, even though most prior evidence comes from metastatic or heterogeneous populations. Alawneh et al. identified clinical frailty indicators, rather than nephrostomy per se, as the principal determinants of short survival after PCN in cancer patients (15). A national UK analysis similarly concluded that high early mortality following PCN in malignancy reflects patient selection for a procedure frequently reserved for those with advanced disease and compromised performance status (14). Guerrero-Ramos et al. reported that only a limited proportion of bladder cancer patients who underwent PCN ultimately received curative-intent treatment, and emphasized that PCN placement without thorough clinical evaluation risks compounding an already adverse trajectory (22). Garg et al. reported that PCN successfully restored renal function in a subset of patients with bladder carcinoma and obstructive uropathy, enabling access to curative or palliative treatment (24). While these findings highlight the potential functional benefit of PCN in selected patients, they do not contradict our results; the survival disadvantage observed in our cohort was not attributable to PCN itself but to the underlying clinical burden of patients who required the procedure. Our findings in a strictly non-metastatic MIBC cohort extend this body of evidence and reinforce the principle that PCN is a clinical context marker, not a standalone prognostic variable.

For metastasis-free survival, treatment modality was the sole independent predictor in multivariable analysis (HR 0.594; 95% CI 0.405–0.872; p = 0.008), with surgery-based treatment associated with a more favorable MFS compared with non-surgery-based approaches. Neither PCN nor hydronephrosis was significantly associated with MFS after adjustment. Treatment modality distribution did not differ significantly between PCN and non-PCN groups in our cohort (50.0% vs. 59.3%, p = 0.139), suggesting that the presence of nephrostomy alone did not systematically preclude patients from surgical pathways. This is somewhat at odds with a multicenter Israeli study reporting that PCN was associated with reduced access to neoadjuvant chemotherapy and suboptimal NAC delivery in MIBC patients (9), though differences in patient selection and center practices may account for this discrepancy. The dominant role of treatment modality in determining MFS underscores the importance of maintaining access to curative surgery in this population, even in the setting of obstructive uropathy.

While the prognostic impact of PCN on survival was attenuated after full adjustment, its association with infectious morbidity was not. Unlike its role as a marker of clinical vulnerability for survival outcomes, PCN may exert a partly causal influence on infectious endpoints, as the indwelling catheter provides a direct route for microbial ascent. PCN was an independent predictor of infection-related hospitalization with an odds ratio of nearly 14 — a magnitude that persisted after controlling for CRP, GFR, albumin, T stage, N stage, and age. This finding reflects a well-recognized clinical reality: nephrostomy tubes provide a continuous portal for ascending infection, and catheter-associated bacteriuria and urinary tract infections are among the most frequent complications in this patient group (10, 11). The high rate of infection-related hospitalizations carries direct consequences for treatment continuity, as infectious episodes can delay or interrupt systemic therapy and increase overall healthcare burden. Emergency department utilization was also markedly higher in PCN patients (median 2 vs. 0 visits, p < 0.001), consistent with the observation by Guerrero-Ramos et al. that catheter-related complications account for a substantial proportion of emergency presentations in this population (22).

A methodological consideration relevant to the infectious endpoints is the asymmetry in urine culture sampling between groups. Positive urine culture was defined based on nephrostomy tube specimens in PCN patients, while midstream urine cultures were obtained under clinical indications in the non-PCN group. This difference in sampling route and frequency introduces a detection bias that cannot be fully corrected in a retrospective design, and microbiological positivity rates should be interpreted with this limitation in mind. The infection-related hospitalization endpoint is less susceptible to this bias, as it reflects a clinically observable event requiring active intervention. CRP emerged as an independent predictor of positive urine culture in multivariable analysis (OR 1.028; 95% CI 1.010–1.047; p = 0.003), consistent with the role of systemic inflammation as both a marker and a driver of infectious susceptibility. Albumin levels were numerically lower in the PCN group (median 35 vs. 38 g/L), and while this difference did not reach statistical significance, hypoalbuminemia is an established risk factor for infectious complications and warrants clinical attention in patients undergoing PCN (9, 11). A similar pattern was observed in the logistic regression model: although lower albumin was associated with higher culture positivity in univariate analysis (OR 0.690; p<0.001), the multivariate estimate attenuated to a non-significant value (OR 1.026; p=0.515), likely reflecting shared variance with PCN and CRP in the adjusted model rather than a true reversal of effect.

Several limitations of this study merit consideration. The retrospective design and the non-standardized nature of PCN placement — based on individualized multidisciplinary judgment — introduce confounding by indication that cannot be entirely eliminated through statistical adjustment. The hydronephrosis-positive subgroup analysis was constrained by the small number of non-PCN patients in that subgroup (n = 14), limiting its statistical power. The sampling asymmetry for urine cultures, as described above, is a further limitation of the infectious outcome comparisons. Institutional variability across two centers and the relatively modest overall sample size are additional constraints. Notwithstanding these limitations, this study provides a systematic characterization of PCN-associated clinical outcomes in a well-defined, non-metastatic MIBC cohort — a population underrepresented in the existing literature, which has largely focused on metastatic or mixed disease settings.

## 5 Conclusions

In this cohort of patients with non-metastatic MIBC, PCN did not emerge as an independent predictor of overall survival after adjustment for ECOG performance status, systemic inflammation, nodal stage, and treatment modality. The survival disadvantage observed in PCN patients on unadjusted analysis appears to reflect the clinical burden these patients carry at baseline — characterized by impaired renal function, systemic inflammation, and more advanced nodal disease — rather than a direct effect of the procedure itself. By contrast, PCN was a strong and independent predictor of infectious morbidity, with markedly elevated rates of positive urine cultures and infection-related hospitalizations that persisted regardless of other patient characteristics. These findings suggest that PCN identifies a clinically vulnerable patient group in non-metastatic MIBC, in whom infectious morbidity is substantial and independent of other disease characteristics. The clinical implication is the importance of careful multidisciplinary evaluation before nephrostomy placement and the proactive management of infectious and systemic consequences thereafter.

## Data Availability

The datasets are not publicly available due to ethical and institutional restrictions related to patient confidentiality. Access to anonymized data may be granted upon reasonable request to the corresponding author, subject to approval by the institutional ethics committee and hospital administration. Requests to access the datasets should be directed to İlkay Çıtakkul, ilkay.citakkul@kocaeli.edu.tr.
